# Can Patient’s Body Weight Represent Body Diameter for Pediatric Size-Specific Dose Estimate in Thoracic and Abdominal Computed Tomography?

**DOI:** 10.25259/JCIS-7-2019

**Published:** 2019-05-24

**Authors:** Supika Kritsaneepaiboon, Suwadee Eng-chuan, Saowapark Yoykaew

**Affiliations:** Department of Radiology, Faculty of Medicine, Prince of Songkla University, Hat Yai, Thailand.

**Keywords:** Body diameter, Body weight, Computed tomography dose index volume, Size-specific dose estimate, Torso

## Abstract

**Objective:**

The objective of the study was to determine whether body weight (BW) can be substituted for body diameters to calculate size-specific dose estimate (SSDE) in the children.

**Materials and Methods:**

A total of 196 torso computed tomography (CT) studies were retrospectively reviewed. Anteroposterior diameter (D_AP_) and lateral diameter (D_lat_) were measured, and D_AP_+D_lat_, effective diameter, SSDE diameter and SSDE_BW_ were calculated. Correlation coefficients among body diameters, all SSDE types and percentage changes between CT dose index volumes and SSDEs were analyzed by BW and age subgroups.

**Results:**

Overall BW was more strongly correlated with body diameter (*r* = 0.919–0.960, *P* < 0.001) than was overall age (*r* = 0.852–0.898, *P* < 0.001). The relationship between CT dose index volume and each of the SSDE types (*r* = 0.934–0.953, *P* < 0.001), between SSDE_BW_ and all SSDE diameters (*r* = 0.934–0.953, *P* < 0.001), and among SSDE diameters (*r* = 0.950–0.989, *P* < 0.001) overall had strong correlations with statistical significance. The lowest magnitude difference was SSDE_BW_−SSDE_eff_.

**Conclusion:**

BW can be used instead of body diameter to calculate all SSDE types, with our suggested best accuracy for SSDE_eff_ and the least variation in age < four years and BW < 20 kg.

**Key Messages:**

Size-specific dose estimate (SSDE) is a new and accurate dose-estimating parameter for the individual patient which is based on the actual size or body diameter of the patient. BW can be an important alternative for all body diameters to estimate size-specific dose or calculate SSDE in children.

## INTRODUCTION

The use of pediatric computed tomography (CT) has grown dramatically in the past decade and the risk of radiation-induced cancers in children is of more concern than in adults. The most commonly used CT parameters for calculating CT radiation dosage are CT dose index volume (CTDI_vol_) and dose length product (DLP).^[1-3]^ However, the CTDI_vol_ is delivered from a specific standard phantom size and does not indicate the actual radiation dose applied to the individual patient, leading to underestimation of the total received radiation dose to children or adults with small body size.^[1-2,4-8]^

Size-specific dose estimate (SSDE) is a new parameter for individual specific patients which was developed by the American Association of Physicists in Medicine (AAPM Report 204).^[9]^ The SSDE is the patient dose estimate with corrections based on the actual size or body diameter of the patient.^[4,9-10]^ There have been several reports examining SSDE in children^[11-15]^ and the combination of measurements (sum of body diameters or effective diameters (D_eff_) is recommended to determine the appropriate SSDE correction.^[11]^ Achieving a patient’s body diameters to calculate SSDE is more difficult than obtaining a patient’s body weight (BW) in routine work, which would make SSDE calculation more simple and rapid. However, only one report has examined conversion factors for pediatric SSDE_BW_.^[16]^ The purposes of this study were to determine whether SSDE based on BW could be substituted for other SSDE values and to compare all SSDE values with CTDI_vol_ among pediatric patients who underwent chest and abdominal CT.

## METHODS

### Patients and study design

The study was approved by our Human Research Ethics Committee. We retrospectively reviewed the imaging records of pediatric patients (<18 years) who underwent intravenous contrast chest or abdominal CT alone or contiguous chest and abdominal CT examinations from October 2011 to October 2016. Of the 2340 studies, 198 were randomly selected by computer, and two studies were excluded due to incorrect CT dose protocols.^[17]^ Finally, 196 studies were reviewed. The demographic data, age, BW, and gender of the patients were collected from the hospital medical records. The patients were categorized into age and BW subgroups. The age subgroups were 0–<5years (*n* = 71), 5–<10 years (*n* = 39), 10–<15 years (*n* = 31), and 15–<18 years (*n* = 55). The BW subgroups were classified according to our institutional practice CT protocol: 4–9 kg (*n* = 19), 10–19 kg (*n* = 70), 20–29 kg (*n* = 22), 30–39 kg (*n* = 15), 40–49 kg (*n* = 26), and 50–64 kg (*n* = 26), >64 kg (*n* = 18).^[18]^

### Definitions, dosimetry, and body diameter measurement

**Figure 1:**
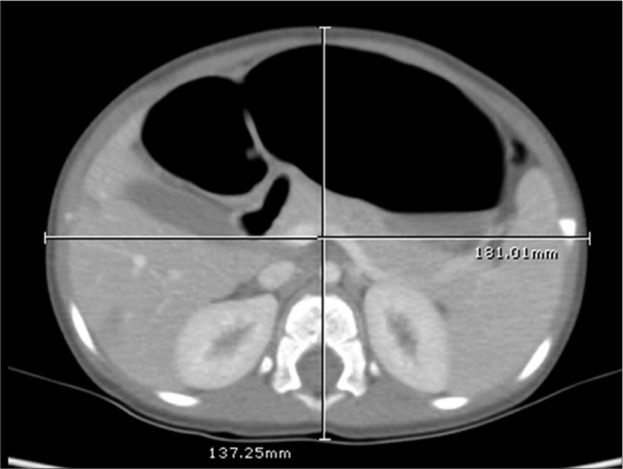
Contiguous chest and abdominal computed tomography (CT) demonstrating the anteroposterior and lateral dimension measurements. A 4-month-old boy weighed 6.1 kg underwent CT scan for tumor surveillance in underlying Langerhans cell histiocytosis.

Anteroposterior diameter (D_AP_) was defined as the skin-to-skin thickness of the body part of the patient at the maximum thickness axial slice image [Figure 1]. Lateral diameter(D_lat_) was defined as the skin-to-skin thickness of the body part of the patient at the maximum thickness axial slice image and/or anterior-posterior dimension localizer image.^[19]^ Anteroposterior plus lateral diameter (D_AP+lat_) was defined as the diameter calculated as AP diameter plus D_lat_. The D_eff_ was calculated as the square root of the AP dimension multiplied by the lateral dimension.^[9]^

The CTDI_vol_ (units: mGy) is the mean radiation absorbed dose to the patient at a given point of scan volume and is defined as weighted CTDI_w_/pitch. The CTDI_vol_ was calibrated using a pencil-shaped ionization chamber with either a dedicated 16-cm or 32-cm diameter polymethylmethacrylate phantom representing the head or a body region, respectively. The DLP was defined as the CTDI_vol_ x exposed scan length. These parameters were displayed on the CT scanner consoles and Picture Archiving and Communication System (PACS). In multiphase-scanning, the CTDI_vol_ of the maximum DLP was used. Only CTDI_vol_ based on 32-cm phantom was included in this study. SSDE were calculated as CTDI_vol_ multiplied by the conversion factor in the table and depended on BW, AP, and lateral and D_eff_ according to the AAPM Report 204 and Khawaja *et al.* study.^[9,16]^ The exact conversion factor for each patient was calculated by the provided equations in the AAPM Report 204.^[9]^

### Data collection

The two CT scanner models used during the study period were a 64-multislice Philips Brilliance CT scanner and a 160-slice Toshiba Aquilion Prime CT scanner. The images were retrieved from a PACS workstation. The body diameters were independently measured by one 13-year-experience pediatric radiologist and one-third year resident training in diagnostic radiology with consensus. The BW, age, dose indices (CTDI_vol_ and SSDE_BW_, SSDE_AP_, SSDE_lat_, SSDE_AP+lat_, and SSDE_eff_), and body diameters (AP, lateral, AP+lat, effective) for each patient were recorded into a spreadsheet (Microsoft Office Excel 2010; Microsoft Corporation, Redmond, WA, USA).

### Statistical analysis

We presented the quantitative parameters involving BW and body diameters (AP, lateral, AP+lat, effective) using median ± interquartile range (IQR) due to non-normal distribution data. Percentage changes between CTDI_vol_ and each SSDE type and the magnitude differences between the SSDE_BW_ and SSDE_diameters_ were calculated.

Correlations among BW, age, dose indices, and body diameter measurements were established with Spearman Rank correlation coefficients (*r*) for the following: Correlations between each body diameter and BW and between each body diameter and age; and correlations among dose indices (CTDI_vol_, SSDE_BW_, SSDE_AP_, SSDE_lat_, SSDE_AP+lat_, and SSDE_eff_) across BW and age subgroups. The power to determine sample size in BW and age subgroups for calculating correlation among dose indices was >0.8. Estimated relationships between median dose indices (CTDI_vol_ and SSDE) and mean BWs were calculated by quantile regression analysis. Differences among the SSDE values were calculated by Wilcoxon Rank sum test. *P* = 0.05 or less was considered to indicate a statistically significant difference. Interobserver variations among the two reviewers were calculated using intraclass correlation coefficient (ICC) values.

## RESULTS

### Demographic data

This study included 196 CT studies from 196 patients, 112 male and 84 female, 72 contiguous chest and abdominal, 66 abdominal and 58 chest CTs. The median BW classified in BW and age subgroups are shown in Table 1. Males had a lower median BW (median [IQR], 18.50 [12.00–47.25 kg]) than females (median [IQR], 25.50 [13.15–46.23 kg]). The largest age subgroup was children 1 day–4 years (*n* = 71, 36.2%) and the 10–19 kg subgroup was the largest BW subgroup (*n* = 70, 35.7%).

**Table 1:** Summary of BW and body diameters by BW and age subgroups.

Parameters	BW^†^ (kg) in median (IQR)			Diameter^†^ (cm) in median (IQR)			
	Total (*n*=196)	Male (*n*=112)	Female (*n*=84)	AP diameter	D_lat_	AP+D_lat_ s	D_eff_
BW subgroup							
Overall	20.40	18.50	25.50	15.42	20.54	35.93	17.81
4–9 kg (*n*=19)	(12.00–47.00) 5.27	(12.00–47.25) 6.10	(13.15–46.23) 4.70	(13.19–19.05) 10.99	(17.55–27.21) 14.04	(31.17–46.66) 25.08	(15.28–22.85) 12.42
10–19 kg (*n*=70)	(4.57–6.83) 13.35	(4.25–7.87) 13.35	(4.70–5.50) 13.55	(10.19–12.53) 13.54	(12.59–14.83) 18.05	(23.09–27.60) 31.47	(11.40–13.47) 15.63
20–29 kg (*n*=22)	(11.07–15.50) 21.75	(11.20–15.87) 21.25	(10.37–15.00) 25.00	(12.81–14.15) 15.00	(17.04–18.85) 20.89	(29.97–32.91) 35.52	(14.84–16.19) 17.56
30–39 kg (*n*=15)	(20.35–25.75) 33.00	(20.45–23.52) 35.50	(20.45–25.75) 32.60	(14.64–15.61) 16.92	(20.18–22.84) 24.33	(35.06–38.08) 41.80	(17.33–18.61) 20.30
40–49 kg (*n*=26)	(30.80–35.75) 45.50	(32.45–36.40) 47.00	(31.02–33.25) 45.00	(15.87–17.94) 17.83	(23.25–25.87) 27.13	(39.90–43.05) 45.32	(19.59–21.26) 22.20
50–64 kg (*n*=26)	(41.40–47.00) 56.80	(44.50–47.50) 58.00	(40.00–46.90) 55.9	(17.18–19.37) 20.81	(25.7–28.27) 29.33	(43.36–47.01) 50.10	(21.29–23.02) 24.65
>64 kg (*n*=18)	(53.50–60.00) 73.45 (65.43–77.33)	(54.00–60.00) 72.75 (65.18–74.33)	(52.70–59.00) 79.15 (71.87–84.52)	(20.06–21.54) 22.24 (21.37–24.04)	(27.83–30.64) 32.42 (29.81–33.52)	(48.05–51.80) 54.52 (52.28–57.43)	(23.62–25.60) 26.62 (25.74–28.32)
Age subgroup							
Overall	20.40 (12.00–47.00)	18.50 (12.00–47.25)	25.50 (13.15–46.23)	15.42 (13.19–19.05)	20.54 (17.55–27.21)	35.93 (31.17–46.66)	17.81 (15.28–22.85)
1 day–4 years (*n*=71)	10.70 (8.75–13.00)	11.20 (9.10–13.50)	9.25 (5.57–11.92)	12.84 (12.24–13.72)	17.05 (15.54–18.14)	30.10 (28.30–31.92)	14.88 (13.93–15.75)
5–9 years (*n*=39)	18.70 (15.75–21.25)	18.70 (17.00–20.85)	17.65 (14.97–22.60)	15.06 (13.76–15.64)	20.35 (18.89–20.67)	35.35 (32.45–36.42)	17.40 (16.05–18.05)
10–14 years (*n*=31)	39.20 (30.25–55.95)	37.00 (30.30–55.40)	42.10 (30.75–56.17)	18.25 (15.73–20.74)	26.37 (23.85–28.67)	45.70 (39.42–48.7)	22.31 (19.25–24.08)
15–18 years (*n*=55)	50.00 (45.50–63.10)	59.00 (47.50–65.15)	47.05 (40.00–53.88)	19.9617.64–21.30)	28.43 (26.35–30.86)	48.10 (44.65–51.75)	23.44 (22.01–25.55)

† Data are expressed as median (IQR), IQR: Interquartile range. BW: Body weight, D_lat_: lateral diameter

### Dose metrics

The overall CTDI_vol_ at 32 cm phantom size was 2.90 (2.88–5.84 mGy) (median [IQR]).

### BW and body diameters across BW and age subgroups

The overall body diameters, AP, lat, AP+lat, and effective, were median (IQR), 15.42 (13.19–19.05), 20.54 (17.55–27.21), 35.93 (31.17–46.66), and 17.81 (15.28–22.85) cm, respectively. The median body diameters across BW and age subgroups are shown in Table 1. The D_lat_ was larger than the AP diameter in all BW and age subgroups. All of the body diameters were in ascending order in both BW and age subgroups. Interobserver agreement using ICC between the two reviewers was excellent (ICC = 0.99).

### Correlation coefficients

Overall and subgroup correlations between body diameters and BW and between body diameters and age are shown in Table 2. The D_AP_, D_lat_, D_AP+lat_, and D_eff_ were strongly correlated to the overall BW (*r* = 0.919, 0.96, 0.935, and 0.943, respectively, *P* < 0.001). The correlations between body diameters and overall age were also strong but less than the body diameter – BW correlations (*r* = 0.852–0.898, *P* < 0.001) [Table 2].

**Table 2:** Spearman’s rank correlation coefficient values for BW, age, and diameter subgroups.

	AP diameter		D_lat_		AP+D_lat_ s		D_eff_	
	Coefficient^†^	*P*	Coefficient^†^	*P*	Coefficient^†^	*P*	Coefficient^†^	*P*
BW subgroup								
Overall	0.919	<0.001	0.960	<0.001	0.935	<0.001	0.943	<0.001
4–9 kg (*n*=19)	0.722	<0.001	0.815	<0.001	0.552	<0.001	0.465	0.039
10–19 kg (*n*=70)	0.638	<0.001	0.718	<0.001	0.77	<0.001	0.706	<0.001
20–29 kg (*n*=22)	0.102	0.651	0.695	0.003	0.46	0.02	0.418	0.052
30–39 kg (*n*=15)	0.522	0.046	0.073	0.795	0.39	0.147	0.450	0.092
40–49 kg (*n*=26)	0.082	0.689	0.496	0.010	0.58	0.001	0.490	0.010
50–64 kg (*n*=26)	0.340	0.097	0.259	0.212	0.34	0.06	0.382	0.059
>64 kg (*n*=18)	0.864	<0.001	0.850	<0.001	0.91	<0.001	0.922	<0.001
Age subgroup								
Overall	0.852	<0.001	0.898	<0.001	0.870	<0.001	0.872	<0.001
1 d–4 years (*n*=71)	0.561	<0.001	0.761	<0.001	0.635	<0.001	0.685	<0.001
5–9 years (*n*=39)	0.078	0.637	0.250	0.12	0.128	0.434	0.128	0.434
10–14 years (*n*=31)	0.150	0.420	0.264	0.15	0.193	0.296	0.189	0.309
15–18 years (*n*=55)	0.181	0.185	0.278	0.040	0.268	0.047	0.24	0.075

AP: Anteroposterior ^†^Spearman’s rank correlation interpretation (*r*): *r*=1 perfectly positive, 0.8≤*r*<1 strongly positive, 0.5≤*r*<0.8 moderately positive, 0.1≤*r*<0.5 weakly positive, 0<*r*<0.1 lowest positive. BW: Body weight, D_lat_: lateral diameter, D_eff_: Effective diameter

The correlations across the SSDE_BW_ and SSDE body diameters in the BW and age subgroups were moderate to strong with statistical significance (*r* = 0.719–0.979, *P* < 0.001 in the BW subgroups and *r* = 0.758–0.965, *P* < 0.001 in the age subgroups) [Table 3]. The correlations across the SSDE body diameters in the BW and age subgroups were strong with statistical significance as shown in Table 4 (*r* = 0.862–1, *P* < 0.001 in the BW subgroup and *r* = 0.872–0.9991, *P* < 0.001 in the age subgroup).

**Table 3:** Spearman’s rank correlation coefficient values for SSDEBW−SSDE diameter by BW and age subgroups.

Parameter	SSDE_BW_−SSDE_AP_		SSDE_BW_−SSDE_lat_		SSDE_BW_−SSDE_AP+lat_		SSDE_BW_−SSDE_eff_	
	Coefficient^†^	*P*	Coefficient^†^	*P*	Coefficient^†^	*P*	Coefficient^†^	*P*
BW subgroup								
Overall	0.934	<0.001	0.951	<0.001	0.942	<0.001	0.953	<0.001
4–9 kg (*n*=19)	0.926	<0.001	0.938	<0.001	0.837	<0.001	0.860	<0.001
10–19 kg (*n*=70)	0.719	<0.001	0.751	<0.001	0.802	<0.001	0.733	<0.001
20–29 kg (*n*=22)	0.899	<0.001	0.933	<0.001	0.940	<0.001	0.935	<0.001
30–39 kg (*n*=15)	0.957	<0.001	0.975	<0.001	0.975	<0.001	0.979	<0.001
40–49 kg (*n*=26)	0.908	<0.001	0.946	<0.001	0.960	<0.001	0.955	<0.001
50–64 kg (*n*=26)	0.949	<0.001	0.939	<0.001	0.953	<0.001	0.956	<0.001
>64 kg (*n*=18)	0.976	<0.001	0.938	<0.001	0.968	<0.001	0.962	<0.001
Age subgroup								
Overall	0.934	<0.001	0.951	<0.001	0.942	<0.001	0.953	<0.001
1 d–4 years (*n*=71)	0.807	<0.001	0.824	<0.001	0.758	<0.001	0.823	<0.001
5–9 years (*n*=39)	0.921	<0.001	0.944	<0.001	0.945	<0.001	0.945	<0.001
10–14 years (*n*=31)	0.927	<0.001	0.865	<0.001	0.922	<0.001	0.899	<0.001
15–18 years (*n*=55)	0.950	<0.001	0.942	<0.001	0.965	<0.001	0.962	<0.001

SSDE: Size-specific dose estimate, BW: Body weight, AP: Anteroposterior diameter, D_lat_: Lateral diameter, AP+lat: Anteroposterior plus D_lat_, eff: Effective diameter. ^†^Spearman’s rank correlation interpretation (*r*): *r*=1 perfectly positive, 0.8≤*r*<1 strongly positive, 0.5≤*r*<0.8 moderately positive, 0.1≤*r*<0.5 weakly positive, 0<*r*<0.1 lowest positive

**Table 4:** Spearman’s rank correlation coefficient values for SSDE body diameters by BW and age subgroups.

Parameter	SSDE_AP_-SSDE_lat_	SSDE_AP_−SSDE_AP+lat_	SSDE_AP_−SSDE_eff_	SSDE_lat_−SSDE_AP+lat_	SSDE_AP+lat_−SSDE_eff_	SSDE_lat_−SSDE_eff_	*P*^‡^
	Coefficient^†^	Coefficient^†^	Coefficient^†^	Coefficient^†^	Coefficient^†^	Coefficient^†^	
BW subgroup							
Overall	0.950	0.989	0.966	0.978	0.979	0.986	<0.001
4–9 kg (*n*=19)	0.989	0.938	0.895	0.997	0.898	0.881	<0.001
10–19 kg (*n*=70)	0.932	0.978	0.961	0.983	0.982	0.967	<0.001
20–29 kg (*n*=22)	0.891	0.969	0.973	0.961	0.997	0.952	<0.001
30–39 kg (*n*=15)	0.982	0.982	0.985	1.000	0.996	0.996	<0.001
40–49 kg (*n*=26)	0.862	0.950	0.956	0.962	0.998	0.959	<0.001
50–64 kg (*n*=26)	0.917	0.976	0.977	0.969	0.9992	0.964	<0.001
>64 kg (*n*=18)	0.953	0.976	0.982	0.988	0.997	0.985	<0.001
Age subgroup							
Overall	0.950	0.989	0.966	0.978	0.979	0.986	<0.001
1 d–4 years (*n*=71)	0.880	0.982	0.895	0.941	0.937	0.973	<0.001
5–9 years (*n*=39)	0.971	0.986	0.990	0.993	0.9991	0.991	<0.001
10–14 years (*n*=31)	0.872	0.956	0.935	0.971	0.977	0.943	<0.001
15–18 years (*n*=55)	0.917	0.972	0.978	0.979	0.998	0.975	<0.001

SSDE: Size-specific dose estimate, BW: Body weight, AP: Anteroposterior diameter, D_lat_: lateral diameter, AP+lat: Anteroposterior plus D_lat_, eff: Effective diameter. ^†^Spearman’s rank correlation interpretation (*r*): *r*=1 perfectly positive, 0.8≤*r*<1 strongly positive, 0.5≤*r*<0.8 moderately positive, 0.1≤*r*<0.5 weakly positive, 0<*r*<0.1 lowest positive. ‡P values for Spearman’s rank correlation coefficient in SSDE_AP_-SSDE_lat_, SSDE_AP_-SSDE_AP+lat_, SSDE_AP_-SSDE_eff_, SSDE_lat_-SSDE_AP+lat_, SSDE_AP+lat_-SSDE_eff_ and SSDE_lat_-SSDE_eff_

### Quantile regression analysis

Quantile regression analysis was used to generate and predict the trends of the median dose indices (CTDI_vol_, SSDE_BW_, and all SSDE body diameters) and BW. The trends of all SSDE values were higher than the CTDI_vol_. The equations to predict dose indices from BW were:

**Table:** 

CTDI_vol_ = (0.09586 × BW) + 1.475231	Equation 1
SSDE_BW_ = (0.104456 × BW) + 4.2285934	Equation 2
SSDE_AP_ = (0.108038 × BW) + 4.465022	Equation 3
SSDE_lat_ = (0.104385 × BW) + 4.915016	Equation 4
SSDE_AP+lat_ = (0.104802 × BW) + 4.753674	Equation 5
SSDE_eff_ = (0.105634 × BW) + 4.698292	Equation 6

### Percentage change

**Figure 2:**
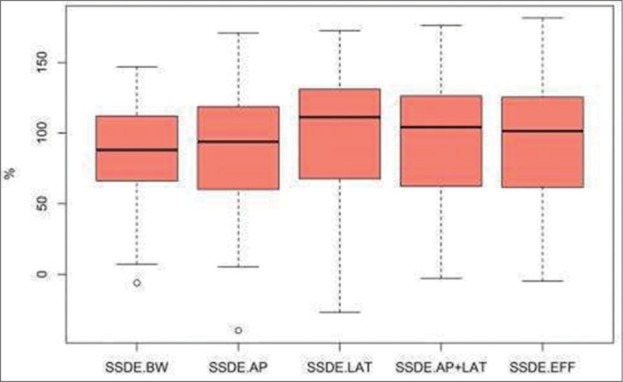
Boxplot percentage change between computed tomography DI_vol_ and SSDE (BW and body diameters).

The percentage change between CTDI_vol_ and SSDE according to the BW and body diameters is shown in the box plot chart in Figure 2. Almost all SSDE values were greater than the CTDI_vol_ values. There was only one patient (0.5%) in which SSDE_BW_ was less than CTDI_vol_ (6%) and this patient weighed >100 kg. In the SSDE diameter group, eight SSDE diameters (SSDE_AP_ =1, SSDE_lat_ = 2, SSDE_AP+lat_ = 2, and SSDE_eff_ = 3) were less than CTDI_vol_, and all of them were maximum diameters in each SSDE diameter subgroup. The percentage change shown as median (IQR) was as follows: (SSDE_BW_−CTDI_vol_)/CTDI_vol_ 88% (66–112%) and range −6–147%; (SSDE_AP_−CTDI_vol_)/CTDI_vol_ 94% (61–119%) with range −39.82–171%; (SSDE_lal_−CTDI_vol_)/CTDI_vol_ 111% (67–99%) with range –27–172%; (SSDE_AP+lal_−CTDI_vol_)/CTDI_vol_ 104% (62–96%) with range −3–176%, and (SSDE_eff_− CTDI_vol_)/CTDI_vol_ 101% (62–94%) with range −5–181%.

### Differences between SSDE_BW_ and SSDE_diameters_

**Figure 3:**
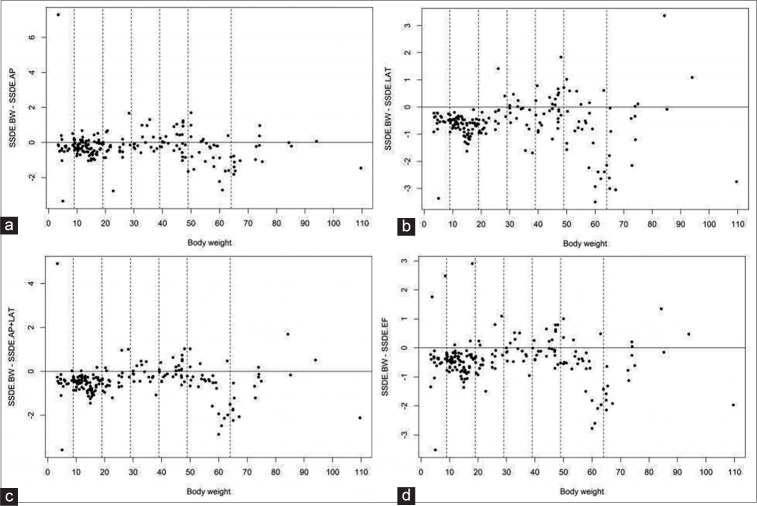
Scatter plots of differences between SSDE_BW_ and each SSDE body diameter by BW subgroups; SSDE_BW_–SSDE_AP_ (a), SSDE_BW_–SSDE_lat_ (b), SSDE_BW_–SSDE_AP+lat_ (c), and SSDE_BW_–SSDE_eff_ (d).

**Figure 4:**
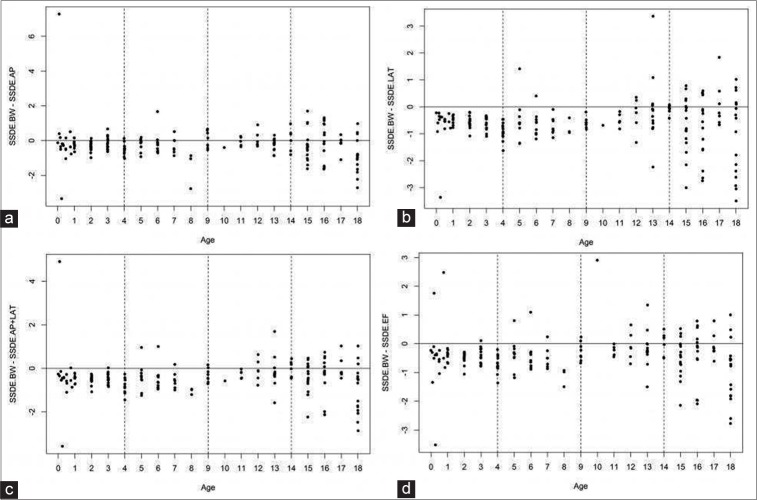
Scatter plots of differences between SSDE_BW_ and each SSDE body diameter by age subgroups; SSDE_BW_–SSDE_AP_ (a), SSDE_BW_–SSDE_lat_ (b), SSDE_BW_–SSDE_AP+lat_ (c), and SSDE_BW_–SSDE_eff_ (d).

The difference between SSDE_BW_ and all SSDE diameters of each patient was not statistically significant in SSDE_BW_−SSDE_AP_ (*P* = 0.3854), and SSDE_BW_−SSDE_AP+lat_ (*P* = 0.09188), and SSDE_BW_− SSDE_eff_ (*P* = 0.1167) except in SSDE_BW_−SSDE_lat_ (*P* = 0.03113) by Wilcoxon Rank sum test. The SSDE magnitude differences between all SSDE_BW_ and all SSDE diameters of each patient were plotted in graphs and categorized by age and BW subgroups [Figures 3 and 4]. The lowest magnitude was the difference between SSDE_BW_ and SSDE_eff._ −4.22–2.91, while the highest magnitude was between SSDE_BW_ and SSDE_AP_ −4.18–7.3. The other magnitudes were −4.31–3.37 for SSDE_BW_–SSDE_lat_ and −4.23–4.91 for SSDE_BW_−SSDE_AP+lat_.

## DISCUSSION

Our study found that all body diameters and overall BW were strongly correlated (*r* = 0.919–0.960, *P* < 0.001). The D_lat_, D_AP+lat_, and D_eff_ in our study had higher correlations with overall BW than with D_AP_, which could be explained by understanding the general growth pattern of children, in which the child’s body grows in the D_lat_ more rapidly than in the AP diameter.^[2]^ The correlations for all body diameters and overall age were also strong but not as high as the body diameter-BW relationships (*r* = 0.852–0.898, *P* < 0.001). However, the study by Kleinman *et al.* found that the predicted individual patient size was not correlated with age.^[2]^

All relationships of SSDE_BW_-all SSDE diameters (*r* = 0.934–0.953, *P* < 0.001) and among SSDE body diameters (*r* = 0.950–0.989, *P* < 0.001) with overall BW and with overall age showed strong and statistically significant correlations [Tables 3 and 4]. The strongest correlations were found in the 30–39 kg subgroup and the 5–9 years subgroup. Another previous study by Khawaja *et al.* reached the same conclusion as our study that BW could be substituted to estimate size-specific dose in children.^[16]^ Another study by Parikh *et al.* also found that BW could be used to estimate SSDE with reasonable accuracy at body width >20 cm.^[14]^

In our study, we could predict the SSDE and CTDI_vol_ using BW from Equations 1–6, while Christner *et al.* study concluded that only CTDI_vol_ increased linearly with patient size (D_AP_ + D_lat_), while SSDE was independent of patient size.^[10]^ Furthermore, almost all SSDE type values (*n* = 189/196, 96.4%) were higher than CTDI_vol_, except for large-sized patients and those weighing >100 kg. Therefore, emphasizing CTDI_vol_ underestimates the radiation dose in most pediatric or small-sized patients and overestimates the radiation dose in large-sized patients.^[2,8,14,20]^

Although the SSDE_BW_ had a statistically significant difference from SSDE_lat_ by Wilcoxon Rank sum test, the magnitude difference between SSDE_BW_ and SSDE_lat_ was still in the acceptable range (within 7% of dose index in diagnostic radiology). The lowest magnitude difference was between SSDE_BW_ and SSDE_eff_, while the highest magnitude difference was between SSDE_BW_ and SSDE_AP_. These results could be explained by considering a study from Brady *et al.*, which found that either an individual AP or D_lat_ measurement alone was less useful than a combination of AP and D_lat_ measurement for SSDE determination.^[11]^ However, all SSDE_BW_–SSDE_diameters_ magnitude differences in our study were still in the acceptable range. The smallest variations of the SSDE differences in all subgroups by age and BW were in the lower BW ranges and younger age groups. In addition, most of the SSDE_BW_ values tended to be lower than the SSDE_diameters_. This implies that the SSDE_BW_ can be substituted for SSDE_diameters_, especially SSDE_eff_, but with caution as the SSDE_BW_ tended to be lower than SSDE_diameters_.

Our study had a few limitations. First, we could not statistically determine correlations between each body diameter and the BW <20 kg and >64 kg subgroups and between each body diameter and the age >4 year subgroups because the power of the sample size in those subgroups was <0.8. We suggest further research should be conducted with increased sample sizes in each subgroup if the study objective is to determine the correlation between body diameters and age or BW subgroups. Second, we did not calculate the SSDE from the water equivalent diameter (Dw), which is a physical parameter based on patient attenuation. In case of patients having high body attenuation, for example, those suffering from mediastinal or intra-abdominal tumors with low to normal BW, the SSDE_Dw_ is more accurate than SSDE_diameter_ to determine the correct patient dose.^[21]^ We suggest further studies including SSDE_Dw_ and clinical indications. Finally, the findings of our study may not be applicable in institutions and hospitals that have automatic software to determine the body measurements and SSDE.

## CONCLUSION

Accurate dose-estimating parameters and size-specific dose indices are important for calculating accurate radiation dosage in the pediatric population. Our study found that the body diameter-BW correlation was stronger than the body diameter – age relationship. This calculation is simple and rapid to perform, and BW can be an important alternative for all body diameters to estimate size-specific dose or calculate SSDE in children. Our findings indicate this method has the best accuracy for SSDE_eff_ and the least variation in ages less than 4 years and BWs < 20 kg.
